# Incidence of infection rate for shunt implantation: the zero % rate is always a myth

**DOI:** 10.1007/s00381-024-06569-4

**Published:** 2024-09-23

**Authors:** Mamadou Diallo, Pierre-Aurélien Beuriat, Alexandru Szathmari, Federico Di Rocco, Pascal Fascia, Carmine Mottolese

**Affiliations:** 1 Department of Pediatric Neurosurgery, Hôpital Femme Mère Enfant, 32 Avenue du Doyen Jean Lépine, 69677 Lyon Cedex, France; 2 Université Claude Bernard, Lyon 1, Villeurbanne, France; 3 Department of Hygene, Epidemiology, Infectiovigilance and Prevention, Hospices Civils de Lyon, France

**Keywords:** Ventriculo peritoneal shun, Pediatric, Infection, Neurosurgery

## Abstract

**Introduction:**

Paediatric CSF shunt infection rate remains a well-known complication that is not only responsible of potentially severe sequels for patients but also for economical expenses. In that study, we questioned if it is possible to attain the zero percent rate of infection that should be the goal of every paediatric neurosurgeon.

**Methods:**

We report our series of patients treated with a CSF device from January the first 2016 to December 31 2018.

**Results:**

In all 147 patients treated for hydrocephalus, the follow-up was of at least of 2 years from the implantation. Antibiotic-coated tubes were always used with a differential pressure valve system. A total of 172 surgical procedures were performed for 147 patients. In the follow-up time period, 4 patients presented a post-operative infection (2.3%). Two infections appeared early after the surgical procedure one after 24 h and the other after 6 days; the other two infections were diagnosed after 53 days and the other after 66 days. The germs responsible of the infections were a *Staphylococcus capitals*, an *Escherichia coli*, a *Klebsiella pneumonia*, and a *Staphylococcus aureus.*

**Conclusions:**

Shunts will always be implanted especially in new-borns and for particular aetiologies of hydrocephalus. To reduce the rate of infection, the best thing to do is to adopt adapted protocols. Our low incidence of infection rate for shunts represent a long history to research preventive factors that helped us to improve our results during the time.

## Introduction

Treatment of hydrocephalus with cerebrospinal fluid (CSF) shunt devices remains a great challenge and a source of worries for neurosurgeons, especially for paediatric neurosurgeons.

Paediatric CSF shunt infection rate remains a well-known complication that is not only responsible of potentially severe sequels for patients but also for economical expenses. The estimated hospital costs associated with shunt infections is 55.000 $ per occurrence in the United States [1].

We already reported our rate of ventriculoperitoneal (VP) shunt infection [2]. In that study, we questioned if it was possible to attain the zero percent rate of infection that should be the goal of every paediatric neurosurgeon. Recently, the HCRN paper reported, with a well definite protocol, an incidence of infection of 5% rate raising the question of the decrease of the infection rate individualizing potentially modifiable risk factors [3].

We report a new series of patients treated with a CSF device and followed with a follow-up of at least two years to verify if we were able to decrease the rate of infection and questioning again on the possibility to reach the zero percent rate of infectious complications.

## Material and method

This is a retrospective monocentric observational study. Inclusion criteria were as follows: a primo implantation or a revision of a VP shunt or other surgical procedure for an internal drainage of CSF anomalies performed in our Service from January 1, 2016, to December 31, 2018.

Patients treated in the same period for a ventricular external drainage were not considered for this study to not consider the increased risk of infection related to the internalization of external drainages.

The study was approved by the local ethical committee. Medical and surgical data were extracted from the hospital medical database. The rate of infection was definite at one and two years after the surgery. The infection was defined as the presence of clinical signs of fever and/or meningitis, the positivity of blood and CSF culture, scar infection or inflammation along the path of the derivation, the presence of abdominal pseudo cysts, and presence of pus or cutaneous dehiscence.

All the patients were regularly followed for at least two years.

Age at surgery varied from two days to seventeen years. We included the patients in six groups of age to highlight a different rate of infectious complications in different chronological period according to the suggestion of the Infection Control Committee (ICC) of our hospital: less than 1 month old (*n* = 21, 12.2%), one to 6 months old (*n* = 22, 12.8%), 6 months to 1 year old (*n* = 17, 9.9%), 1 to 4 years old (*n* = 47, 27.3%), 4 to 10 years old (*n* = 23, 13.4%), and older than 10 years (*n* = 42, 24.4%).

The protocol used was revised when the Paediatric Neurosurgical Service was transferred in the New Paediatric Hospital (2016), and it was conceived according the protocol proposed by Choux and transformed during the time in function of our own experience and expertise.

The protocol used was based on the following steps:

- A shower and a shampoo before the operative room realized with polyvidone scrub or chlorhexidine scrub for patients younger than three months old, for the skin preparation.

The shower and shampoo were realized with the old protocol three times and with the consensus of the CLIN only the evening before the surgery and early in the morning before to leave for the operative room.

For patients allergic to the polyvidone and to the iodine derivate, a protocol with hibiscrub and biseptine was used. Patients operated in emergency were submitted at least to a shower and a shampoo before the surgical procedure.

In the operative room, the access was limited to the anaesthesiologist and the anaesthetist’s nurse, two neurosurgeons, two ancillary nurses, one for the instrumentation, and the other for the circulation.

At the installation of the patient on the operative table, with the patient intubated, a new shampoo was realized by surgeons. Hairs were not shaved except for a little space for the incision. The operative field was delimited with a sterile adhesive field before the pre-asepsis with the antiseptic products.

A cephalosporin of second generation was injected twenty minutes before the surgical incision, and in case of re-implantation or of allergy, vancomycin was used to prevent infectious complications while before they were administrated for forty-eight hours.

After the asepsis of the patients’ sticky fields were used to delimitate the operative field, an incision drape with or without iodine products, in function of the age of patients, was applied.

Two pairs of gloves were used, and the outer pairs were changed after the dressing of the patient, at the moment when the body of the valve was implanted and at the moment of the ventricular tab and finally at the closure of the surgical cicatrix.

A no touch technique was normally adopted for the manipulation of the device.

At the end of the procedure, a shampoo was realized with antiseptic soap by the nurse and the cicatrix protected after the application of yellow betadine or biseptine with pinching.

Antibiotic coated tubes of the Codman company were used, and in the main number of cases, a programmable opening pressure valve (CODMAN Medos) was used.

In the same period, thirty patients not considered for this study were operated for an external ventricular drainage for bacterial meningitis, traumatic intracranial hypertension, intraventricular haemorrhage for AVM malformation rupture, and aneurism rupture.

### Care in the service the day after the surgery

The day after a new shampoo and a shower were realized and the surgical cicatrix left at the air after the application of antiseptic solution.

The surgical scars were cleaned with a local application of antiseptic twice day for two or three days.

The patients were discharged after four days with the prescription to remove the cutaneous stitches, after a week, by a nurse or in our service.

## Results

In all, 147 patients treated for hydrocephalus the follow-up was of at least of two years from the implantation and four infections were recordered (2,7%). Sixty percent of patients were males and 40% females.

The aetiology of hydrocephalus was in 44% of cases congenital (stenosis of aqueduct, haemorrhagic hydrocephalus, meningitis), tumoral in 17% of cases, malformative (myelomeningocele) in 4% of cases, and of different aetiologies in 22% of cases. Thirteen percent of patients were treated for an arachnoid cyst or to drain a subdural effusion uni or bilateral.

In tumoral hydrocephalus, the tumours treated were medulloblastomas, pilocytic astrocytomas, ependymomas, choroid plexus papillomas, and craniopharyngioma.

In the group of different aetiologies, six patients were treated for a sub-dural hematoma, six patients for a post-infectious meningitis developed at distance, four patients after an intracranial surgery complicated with a hydrocephalus, and four patients for a post-traumatic hydrocephalus. For thirteen patients treated, the aetiology of CSF troubles were not established.

In 35% of patients, the implantation of shunt was realized before the age of one-year-old.

Eighty-four patients (57%) were treated for a first implantation and the others 63 patients (33%) for a revision related to a mechanical problem or a systematic lengthening of the distal tube in relation with the growth of patients.

Two infections appeared early after the surgical procedure one after 24 h and the other after 6 days; the other two infections were diagnosed after 53 days and the other after 66 days.

The germs responsible of the infections were a *Staphylococcus capitals*, an *Escherichia coli*, a *Klebsiella pneumonia*, and a *Staphylococcus aureus*. The infection rate was of 2,3% considering 172 surgical procedures for 147 patients.

For 30 patients, not considered for this study and operated for an external ventricular drainage for different aethiologies, the rate of infectious complication was 3.3%.

### Cases infected

#### Case 1

A 3-month-old patient with an history of prematurity was treated with a shunt for a post-haemorrhagic hydrocephalus. Two months after the implantation, the child was hospitalized for a febrile state and a distension of the abdominal wall. An abdominal CT-scan shoved an abdominal cyst. A lumbar puncture for CSF study and blood examination and blood culture confirmed the infection. The externalization of the shunt was decided and an antibiotic treatment was prescript for 3 weeks. At this moment, after that a CSF study showed its sterility, a new shunt was implanted changing the side of implantation, and the patient discharged after a week with a good evolution.

#### Case 2

A 2-year-old child with the diagnosis of a Dandy Walker syndrome arrived in our hospital at the emergency service presenting signs of intracranial hypertension.

In his history, we discovered a vesicoureteral reflux of high grade treated with a vesicotomy in the country of origin and multiple urinary tract infections.

A surgical indication was retained, and a ventriculocisternostomy of the third ventricle was realized tied to a cysto-peritoneal shunt for the presence of a large cyst at level of the posterior fossa.

The surgical procedure was realized without problems and the patients discharged after the ablation of the stitches after a week.

Three months later, a fever was the reason for an emergency consultation.

A lumbar puncture and urine culture showed the presence of an *Escherichia coli*, and the biological diagnosis of meningitis, on the CSF study, pushed to the externalization of the shunt. A treatment with adapted antibiotics was started, and a new internalization of the system was possible, changing the side, 3 weeks later with a good evolution.

#### Case 3

A 3-month-old baby arrived from the African continent with a severe congenital triventricular hydrocephalus. The baby was malnourished with infected bedsores. The baby was awake but with a permanent sunset ocular sign and an important increase of the head circumference. Emesis were also present. No fever was reported.

A ventriculoperitoneal shunt was decided to stop the evolution of the hydrocephalus and to avoid the progression of a papillary oedema discovered to the ocular fundus examination. We decided to implant the shunt directly to reduce the costs for the family of a longer hospitalization after 3 days of preparation as outpatient during which local treatment with antiseptic products and local antibiotics on the bedsores was realized.

The day after the surgical procedure fever and vomiting appeared justifying infectious investigations. The presence of germs in the CSF culture confirmed the clinical diagnosis of meningitis that obliged us to the externalization of the shunt. The germ individualized was a *Klebsiella pneumoniae*.

After a 3-week treatment with adapted antibiotics and a program to improve the malnourished condition, a new shunt was planned, and finally the patient was discharged with a good evolution. The social protection was obtained, and the family is living now in France, and he is now regularly followed in our service and has a good neurological evolution.

#### Case 4

An 11-year-old child was hospitalized for an intracranial hypertension related to a posterior fossa tumour and an acute triventricular hydrocephalus.

The patient was operated in emergency for an endoscopic ventriculocisternostomy of the third ventricle and, 48 h after, for the removal of the posterior fossa tumour, a pilocytic astrocytoma.

The patient was discharged, but 3 weeks after, he presented again clinical signs of intracranial hypertension with a swelling from the posterior fossa scar due to a pseudo-meningocele. A CT-scan shoved an acute hydrocephalus that was treated with a ventriculoperitoneal shunt.

Two days after, the patients presented a suppurative skin fistula of the posterior fossa cicatrix and signs of meningitis. A lumbar tab confirmed the diagnosis of meningitis, and the germ isolated was a *Staphylococcus aureus*. A surgical procedure was programmed to clean the surgical field associated in the same time with externalization of the shunt and an adapted antibiotic therapy.

The antibiotic treatment was effective, and after the sterilization of the CSF, a new shunt was realized 3 weeks after changing the side with a good evolution.

## Discussion

The analysis of our cases infected pointed that in two patients, there was a history of multiple infections, multiple urinary tract infections in a case, and infected bedsore in a patient with a malnutrition state. For another patient, an infection at level of the operative field for a previous posterior fossa tumour surgery responsible of the contamination of the shunt was inserted for a post-operative hydrocephalus. A patient was treated for an intraventricular haemorrhage at birth.

The infection rate of shunt implantation for treatment of hydrocephalus remains a challenging problem for all paediatric neurosurgeon’s world wild. The respect of strict protocols for the preparation of patients for the surgical procedure and for the surgery has been stressed, with an impressive reduction of the infection rate, as reported by Schaffzin [4]. For this author, the adoption of a strict “surgical” protocol improved of 79% the infection rate risk for all paediatric procedures [4].

Standardized protocols reduced the risk of infection due to implantation of baclofen pump of 50% [5] and also, for surgery of scoliosis, a significant reduction from 8.6% to only 2% was observed [6].

The Hydrocephalus Clinical Research Network (HCRN), also with a standardized protocol, reported a reduced shunt infection rate [3, 7]. With the participation of 9 centres, the rate of shunt infection was of 5.1% within 12 weeks, using of tubes coated with antibiotics and the irrigation of the surgical scars with vancomycin. The study seems us to have a short follow-up for the control of the infection rate because the length of the post-operative monitoring should be of at least 12 months after the implantation of foreign material [3].

Our rate of infection for shunt implantation after a 2-year follow-up was of 2.3% inferior to the infection rate reported by the HCRN [8].

Our protocol was derived from that of Choux [9] and discussed by the ICC of our hospital that takes in care the prevention and the quality of improvements against infections acquired in our hospital.

The rate of infection in treatment of hydrocephalus with shunt represents an old problem, and, as reported by Bayston [10], it varied from 0 to 30%.

In our hospital during the 1980s, the rate of shunt infection was of 7% very high.

At this period, the paper of Choux [9] was preconizing a zero percent infection rate that pushed us to modify it for treatment of hydrocephalus with shunt, and so we changed adopting also on new devices:

- Three antiseptic washings
- Two shampoos before the surgical procedure
- The procedure realized early in the morning, the youngest patient operated on first, no more than three shunt operations/day except emergency
- Antibiotic prophylaxis with cefuroxime for 48 h or with vancomycin for allergic patients or for re-implantation

With this protocol the rate infection fell to 4.2%, and the study of 30 factors of risks for infection showed that the ratio between patients operated, and the procedures realized was not a risk factor on the contrary of aetiology of hydrocephalus and age of patients [2].

But these two risk factors had a weaker impact as responsible of infections after the adoption of a strict protocol with the adoption of three showers and shampoos for patients before the operative room and the respect of a strict attitude in the operative theatre.

A significant risk factor was the material of the shunt used, and we changed the type of shunt, the old Holter device with a fixed opening pressure, for a more modern shunt for that epoch, the paediatric CODMAN Medos pressure reglable shunt, and in the same time the implant of antibiotic-coated tubes. With these resolutions, the infection rate decreased to 1.4% for patients and 1.3% for surgical procedures [2]. In this period, we asked the question if it could be possible to join the zero percent incidence of infection for shunt device for treatment of hydrocephalus. It was evident that the strict application of a protocol and the use of new materials of shunt and antibiotics coated tube allowed us to reach a lower rate of infection. The measures taken had a strong impact when from a higher rate of infection; it should be desirable to join a lower rate of infection decreasing of two, three, and four percent, but their efficiency is less evident to demonstrate when the rate of infection is already low.

To demonstrate the efficacy of tubes coated with antibiotics in service with already a low rate of shunt infection and to further reduce the incidence of infections are necessary for a great cohort of patients and very long studies, eventually cooperative studies, with a homogeneity of factors to be respected by the participants but also with a homogeneity of human factors more difficult to manage.

An important point to evaluate the infection rate is the duration of the follow-up that for us have to be of at least one year as reported in our study.

Our results are better than those of the HCRN considering that our rate of infectious complications is reported after a follow-up of at least 1 year for each patient and without antibiotics irrigation of surgical incisions but only using a prophylactic antibiotic treatment injected 20 min before the surgical procedure. We are aware that our series is smaller than that of 4913 shunt procedures reported by the American Consortium for Hydrocephalus, but if we consider single centre, the infection rate reported for treatment of hydrocephalus with shunts in single centre is higher because 5.1% represents an average with centres’ with a more important rate [3].

An important point of discussion is the length of the follow-up that for us have to be at least of one years to record infection that appear later.

Currently in France, the French Society of Infectious Pathologies of French Language (SPILF) and the French Society for Anesthesia and Revival (SFAR) preconize a surveillance of three months for the infections of the surgical site also for the implantation of device. We think that a duration of 3 months is too short and can mask infections that can appear later reducing artificially the true rate of infectious complications for shunt, and so we decided to report the rate of infection after a follow-up of at least one year.

The HCRN considered a follow-up of only 6 months for the infection rate [3]. This short period of control represented also the main criticism concerning the paper of Choux about the incidence of zero% infection potentially excluding the infections appearing later [9].

The length of the follow-up and the use of a same methodology during at least 10 years and the same surgical team during the same period of the study represent a point of force of our study. We did not use antibiotics for irrigation of the surgical wounds because they can favourite resistance and an inflammatory reaction at the origin of a possible infectious contamination and a delayed healing process.

Complex pathologies are associated with a higher rate of infection, but the strict application of protocols modulate their responsibility as for other factors like age and the aetiology of hydrocephalus [3, 9].

Kulkarni reported an increased incidence of infection in child under 6 months, but we did not find this difference as also reported by Faillace [11], and this fact was correlated with the better preparation of patients for the operative room with showers and shampoos that reduce the risk of operative contamination [2, 9]. Another explanation for a low incidence of infection in very young patients, as observed in our cases, is the less important power of contamination with the hands of the surgical scars.

Pirotte reported no infection with a protocol with limited skin manipulation, no touch technique, and a limited duration of the surgery and a systematic use of prophylactic antibiotics [12].

The length of hospitalization was also incriminated as responsible of the increased rate of infection, but also for this factor, we did not find a significant influence: The median duration of hospitalization for shunt surgery was 4 days varying from 2 to 7 days when the stitches were retired in the service before the patient’s demission.

The surgical scars after the first 24 h were left without pinching and treated twice a day with application of an antiseptic solution as for example povidone iodine until the removal of the stitches.

The use of impregnated tubes with antibiotic reduced the infection rate significantly [13] as we already reported [2] even though others authors did not observed the same results [14].

Coated tubes with antibiotics decreased the rate of infection, but they were not able to contribute to the disappearance of infections. Coated tubes with antibiotics promoted the modification of the bacterial flora responsible for infection. While before their use the most common organisms were the coagulase negative staphylococcus, *Staphylococcus epidermidis* and the *Staphylococcus aureus* [15], after their introduction the presence of phoecal germ and environmental germs increased modifying the spectrum of more traditional germ.

The antibiotic coated tubes release antibiotics locally for a duration of quite 2 months, so a short follow-up can neglect the appearance of late infection after 3 months, 6 months or after 1 year.

The presence of chronic complex condition, as reported by Feudtner [16], increases the infection rate, but the necessity to control the increased intracranial hypertension can push to treat hydrocephalus with a shunt instead of waiting for better general condition for surgery.

This situation was found in one of our cases when the suspicion of a possible infected patient with a malnutrition condition should have pushed us to realize a temporary external drainage to permit, with antibiotics to sterilize the suspected infectious state, and with a program of nutrition, to implant the shunt in a more favourable condition.

The nutritional state and the economic social level represent a significant factor risk because it conditions the development of the immunity system predisposing to an increased incidence of infection in surgery for VP shunt [17].

In an emergency, also with an established protocol, surgery for implanting a shunt can be source of mistakes because it became difficult for all the actors, at each level of the chain, to respect all the steps of the protocols explaining, in our opinion, why it is very difficult to attain zero percent of infections related to the shunt surgery. In paediatric neurosurgery, an acute hydrocephalus represents a real emergency situation, and it is difficult to postpone surgery but every effort has to be employed to maintain, in each circumstance, a high level of attention in all the steps of the procedure because each detail is important to avoid mistakes.

It is of paramount importance to use a flawless surgical technique, the respect of a strict protocol for preparing patients for the operative room, and a strict protocol by the surgical team when the surgical procedure starts. The dressing of patients has to be done with rigor, as attention needs the surgical procedure and, above all, at the end of the procedure, when the drapes have to be removed attention is necessary to cover the surgical scars to avoid that the surgical drapes, in contact with the floor, can touch the skin contaminating the scars. This is an important step for all surgical procedures but especially for shunt surgery.

Surgery for shunts is and remains, in our opinion, very difficult because if everyone can put a shunt in place, to put a shunt that works and that it shall not be infected is not given to all surgeons even if they are very skilful. The consequence of a shunt that does not work and that is infected can be catastrophic for patients and mainly for small babies that can have the brain completely destroyed after a ventriculitis or a severe meningitis.

Zero percent of infection should be the aim of all neurosurgeons and of all paediatric neurosurgeons but, also if the perfection does not exist in our world, to reduce the rate below two percent should be possible adapting the protocols adapted to the local conditions and being very severe for the preparation of patients that go to the operating room respecting well the program of prevention to decrease the risks of infectious contamination.

Our experience and others of literatures [2, 13, 18] have shown that the antibiotic-coated tubes lower the infection rate reducing also the costs related to the care of hydrocephalus.

In the BASIC study reported by Mallucci [18], the expenditure were calculated at level of 135753 £ recommending, in consequence, the use of antibiotic coated tubes for all patients undergoing first time a ventriculoperitoneal shunt surgery.

We agree with this statement, and we believe that the higher cost related to the material permits to realize a great saving expenditure important not only for each patient but also for all the society.

The HCRN reported the different efficacy of antibiotics and antiseptic solutions used for the wound irrigation but reported also that some products were retired by the US FDA for the risks of nephrotoxicity and anaphylactic reactions [3, 7]. We think that the use of antibiotics for wound irrigation can be responsible of the selection of resistance and also of toxic reaction [8].

Concerning the risk related to the shunt revision, we found that a revision for mechanical problems does not expose to an increased rate of infection.

In our study, only 27 patients (18%) were operated for a re-implantation of the shunt. Of these 27 patients, if we exclude the four patients that presented an infection and that needed a reimplantation, 5 were operated for a severe post traumatic hydrocephalus (2 cases), and for an intraventricular haemorrhage of the new-born (3 cases).

In the other 18 cases, the shunt was revised for mechanical problems related to the body of the shunt, or to a disconnection at level of the ventricular catheter or between the body of the shunt and the peripheral catheter or for the fracture of the distal catheter migrated in the peritoneal cavity, while in the other cases, it was a programmed lengthening of the distal tube in growing patients.

Comparatively, the rate of infection for ventricular external derivation was of 3.3%, and we think that this acceptable rate of infection was in relation with the fact that for all patients, when possible, the surgical procedure was realized in the operative theatre with the same protocol for elective shunt procedures.

## Conclusions

Also, if the old statement says that the best treatment for hydrocephalus is not to shunt [19] and also if ETV seems a good technic to cure hydrocephalus mainly for tumoral or congenital obstructive aetiology, patients will continue to need the implantation of shunts. The shunts will be implanted especially in new-borns and for particular aetiologies of hydrocephalus and consequently to reduce the rate of infection; the best thing to do is to adopt adapted protocols keeping in mind that surgery for shunt remains the most difficult surgery for the speciality of neurosurgery and to operate patients always with some concentration to avoid diversion that can conditionate the final result. Our low incidence of infection rate for shunts represent a long history to research preventive factors that helped us to improve our results during the time.

We think that when we speak of the infection rate for shunt surgery, it should be imperative to consider at least one year of follow-up. If this delay of follow-up is respected, a rate of shunt infection between one and two percent can be acceptable also if, it is important, to pursue efforts to approach zero percent rate that seems unapproachable, until now.

## Data Availability

No datasets were generated or analysed during the current study.
